# Analysis of risk factors for perioperative complications in spine surgery

**DOI:** 10.1038/s41598-022-18417-z

**Published:** 2022-08-23

**Authors:** Nicole Lange, Thomas Stadtmüller, Stefanie Scheibel, Gerda Reischer, Arthur Wagner, Bernhard Meyer, Jens Gempt

**Affiliations:** Department of Neurosurgery, Klinikum rechts der Isar, Technical University Munich, Ismaningerstraße 22, 81675 Munich, Germany

**Keywords:** Pathogenesis, Risk factors, Signs and symptoms

## Abstract

Complications in spine surgery can arise in the intraoperative or the immediate postoperative period or in a delayed manner. These complications may lead to severe or even permanent morbidity if left undiagnosed and untreated. We prospectively interviewed 526 patients out of 1140 patients who consecutively underwent spinal surgery in our department between November 2017 and November 2018 and analysed the outcome and complication rates. A 12 months follow-up period was also adopted. We analysed the patients’ clinical characteristics, comorbidities, surgical management, survival rates, and outcomes. Risk factor analyses for the development of complications were also performed. Patients’ median age was 67 years (range: 13–96). The main diagnoses were as follows: degenerative in 50%, tumour in 22%, traumatic fractures in 13%, infections in 10%, reoperations in 3%, and others in 2%. Surgeries were emergency procedures (within 24 h) in 12%. Furthermore, 59% required instrumentation. The overall postoperative complication rate was 26%. Revision surgery was required in 12% of cases within 30 postoperative days (median time to revision 11 days [IQR 5–15 days]). The most frequent complications included wound healing disorders, re-bleeding, and CSF leakage. Thereby, the risk factor analysis revealed age-adjusted CCI (p = 0.01), metastatic tumour (p = 0.01), and atrial fibrillation (p = 0.02) as significant risk factors for postoperative complications. Additionally, postoperative KPS (p = 0.004), postoperative anaemia (p = 0.001), the length of hospital stay (p = 0.02), and duration of surgery (p = 00.002) were also identified as associated factors. Complication rates after spinal surgeries are still high, especially in patients with metastatic tumour disease and poor clinical status (KPS), requiring revision surgeries in several cases. Therefore, specific risk factors should be determined to carefully select surgery groups.

## Introduction

Complications in spine surgery can emerge in the intraoperative or the immediate postoperative period or in a delayed manner post discharge. These complications may lead to severe or even permanent morbidities if left unrecognized and untreated^1–5^. They may also extend hospital stay, lead to higher readmission rates, and cause chronic pain. Therefore, an analysis of risk factors to develop strategies for their prevention and to ensure the maintenance of high quality of medical care is essential.

In light of an increasing number of older patients with a higher number of comorbidities often being referred to tertiary spine centres, surgeons are forced to balance the risks associated with operation against the potential benefits for their patients. This highlights the importance of evidence-based operative and non-operative therapies and has led to several clinical studies analysing complication rates, risk factors, and outcomes of the applied techniques in different departments. Thus, due to the heterogeneity in spine surgeries, data on complications after spinal surgery is rare.

The aim of this study was to analyse complication rates after spinal surgery in a representative consecutive cohort in a tertiary spine centre for a period of one year and critically report the risk factors associated with the surgery.

## Materials and methods

We prospectively interviewed 526 patients out of the 1140 patients who had consecutively undergone spinal surgery in our department between November 2017 and November 2018 and analysed their surgical outcomes and complication rates. A 12 months follow-up period was adopted as well. We analysed the patients’ clinical characteristics, comorbidities, surgical management, overall survival rate, and outcomes.

Clinical data of the patients included age, sex, main diagnosis, date, time, and length of surgery, previous spine surgeries, count and location of spinal levels included in the surgical field, type of instrumentation, and pre- and postoperative laboratory values. Additionally, the lowest intraoperative haemoglobin levels, body temperature and intraoperative blood pressure, count of transfusions administered within 30 days of perioperative care, length of hospital stay, emergent versus non-emergent surgery were also recorded. The occurrence of complications including type, date, and the necessity of revision surgery within the follow-up period was also assessed.

Within this work, we defined and selected the complications accodring to the proposals of the german spine registry (DWG). This is a nationwide register of all spine centers giving them the possibility to record indication, operative technique, and complications of their spine surgeries. The registry was built up based on the american spine registry. We grouped the complications into intraoperative and postoperative, as well as systemic and surgical. Intention was to enable future comparison to any european or american analysis using the same categories.

Complications were grouped into intraoperative complications (screw revisions due to mispositioning, accidental Dural lesions, injury of the vertebral artery, premature termination of surgery due to high blood loss or cardiopulmonary instability, accidental rhizotomy, cement leakage) and postoperative complications (wound healing disorders, rebleeding, CSF leakage, urinary tract infection, novel back pain, abscess/spondylodiscitis, material dislocation, motor deficit, screw mispositioning, novel leg pain, persistent stenosis, pulmonary embolism, renal insufficiency, adjacent segment degeneration, pneumonia, bloodstream infection, new dysesthesia, drain demolition, ileus, cardiac event, dysphagia, and psychiatric disorders).

The recorded comorbidities included American Society of Anaesthesiologists (ASA) classification, hypertension requiring medication; diabetes mellitus; history of chronic obstructive pulmonary disease; preoperative pneumonia; cardiac comorbidities including newly diagnosed or worsening congestive heart failure during a 30 day period before surgery, myocardial infarction 6 months before surgery, angina, and a history of cardiac surgery or percutaneous coronary intervention; peripheral vascular disease including revascularization and rest pain; liver disease defined as ascites within 30 days before surgery or oesophageal varices; renal dysfunction defined as acute renal failure within 24 h before surgery or as dialysis dependence (≤ 2 weeks before surgery); history of paralysis (hemiplegia, paraplegia, or quadriplegia); malignant neoplasm (presence of disseminated cancer or recent history of radiation therapy or chemotherapy); alcohol and nicotine abuse. Thus, the calculation of the Charlson Comorbidity Index (CCI)^7^ and Diagnosis Count (DC)^8^ was performed on the patient data.

The questionnaires included a visual analogue scale (VAS), short-form (SF)-12 and Oswestry disability index (ODI), as well as subjective life quality recorded on a scale from 0 to 100. They were either filled by the patients themselves, or by the interviewing person during direct interviews or telephonic interviews.

**Figure 1:**
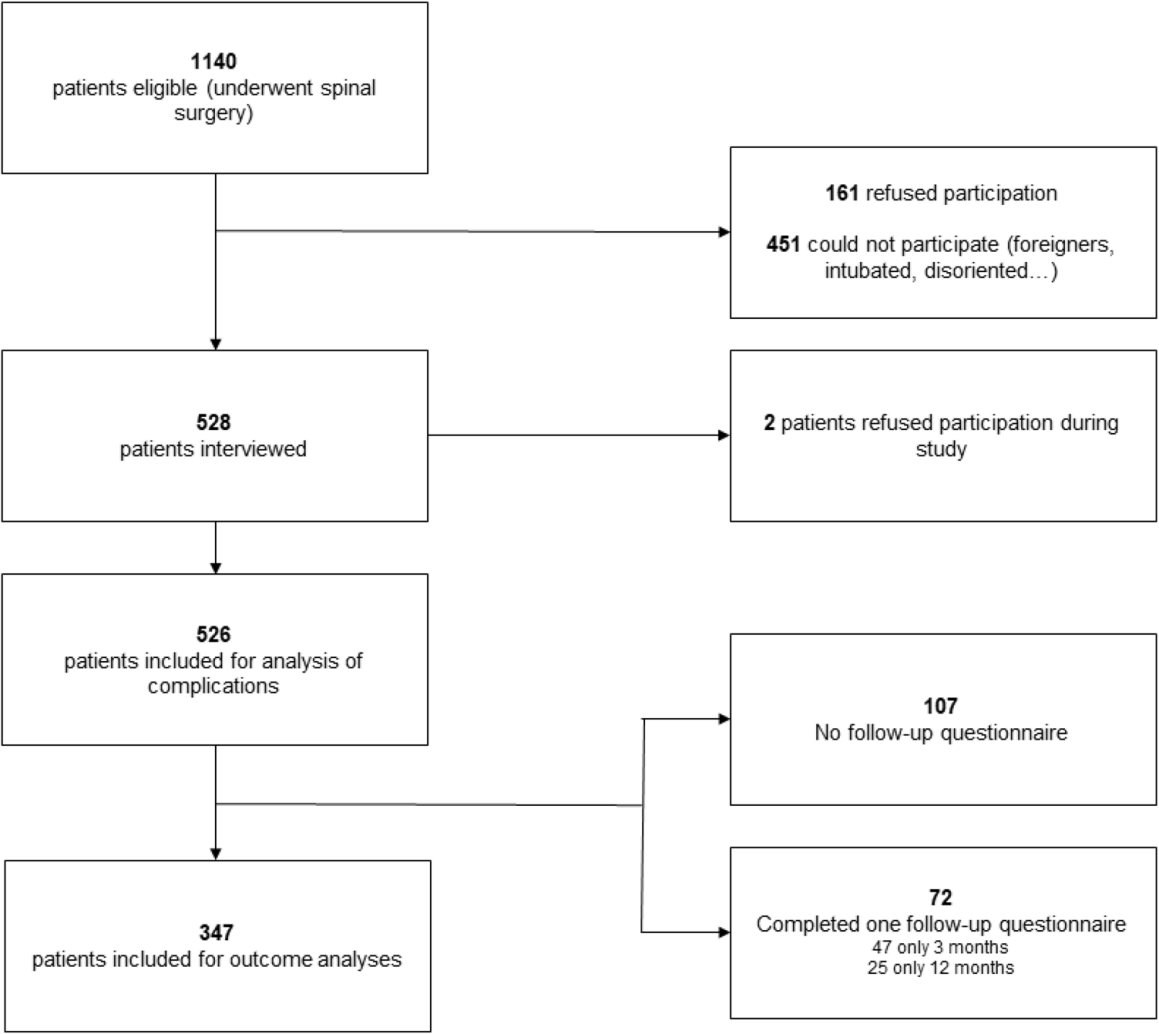
Flowchart showing patient inclusion and exclusion throughout the study.

We undertook calculations using the SF-12 and ODI at the time of admission and during the follow-up period (after 3 and 12 months). Mortality rates during the follow-up period were also assessed. The present study was approved by the local ethics committee (local ethics committee of the technical university of munich) and performed per the ethical standards established by the 1964 Declaration of Helsinki and its later amendments^9^ (Clinical Trial Registration Number: 205/18S). Since the inauguration of the DWG spine registry in 2012, every patient is asked to provide informed consent before surgery to allow the integration of the anonymous data into the registry. Additionally, patients were asked to sign an informed consent form to allow the assessment of their data and clinical status before and after surgery since they were included in this study. A flowchart with the inclusion and exclusion criteria is presented in Fig. 1.

Overall, during this period, 1140 patients underwent spinal surgery in our department and were therefore suitable for this study. Of them, 161 patients refused to respond to the questionnaires, 451 patients did not participate because of other reasons (being foreigners, disoriented, or intubated patients, cancellation of further surgery), leaving 526 patients included in the analysis of complications. For the outcome analysis, only patients who completed the 3- and 12 months of follow-up assessments were included. In this study, a total of 347 patients (66%) were included.

Statistical analyses, including descriptive data analyses, were performed using 3.6.2 (R Core Team, www.r-project.org). Associations between all the assessed binary variables were analysed using the two-tailed chi-square test or t-tests for categorical and continuous variables respectively. To identify the potential risk factors for the outcome changes, a multivariate logistic regression analysis was undertaken. For all analyses, a difference with an error probability of less than 0.05 was considered statistically significant. Odds ratios with 95% Cis were calculated. The descriptive statistics for demographic variables were generated with means and SDs or medians with interquartile ranges as appropriate.

## Results

### Patient population

Clinical characteristics are presented in Table 1.

**Table 1:** Demographic and clinical characteristics of patients.

**Age (years; median range)**	67	13–96
Distribution (no.; Percent) < 30 years	20	4%
30–39 years	34	6%
40–49 years	40	8%
50–59 years	85	16%
60–69 years	123	23%
70–79 years	148	28%
≥ 80 years	76	14%
**Sex (no.; percent)**		
Female	234	44%
Male	292	56%
**Main diagnosis (no.; percent)**		
Degenerative	262	50%
Tumour	116	22%
Fracture/Trauma	69	13%
Infection	50	10%
Others	12	5%
**Comorbidities (no., percent)**		
Hypertension	227	43%
Diabetes	80	15%
Smokers	83	16%
Alcohol abuse	78	15%
Metastatic tumour	87	17%
Anticoagulant medication	160	30%

Between November 2017 and November 2018, 1140 patients underwent spinal surgery in our department due to various pathologies. Of them, 526 patients (292 males, 234 females) were included in this study analysing complications. The patient’s median age was 67 years (range: 13–96).

General characteristics of the patient population indicated it to be overweight with a mean BMI (Body Mass Index) of 27 (range: 12–52). The mean ASA score was 2, reflecting a moderate but definite systemic disturbance. Mean DC (Diagnosis Count) was 2 and range 0–12, leading to a mean CCI of 2 (range: 0–17) highlighting the likelihood of mortality within a 10-year period in 48% cases.

Of the subjects, 16% were smokers and 15% admitted to alcohol abuse (on a daily basis). Regarding co-medications, 30% were under anticoagulant medication, which was continued perioperatively in 9%.

The main diagnoses were degenerative (50%), tumour (22%), traumatic fractures (13%), infections (10%), and others (5%).

### Surgical procedures

Emergency surgeries (within 24 h) were performed in 12% of the patients with a mean duration of 142 min (range: 11–440). Operations comprised 3 vertebral segments on average and were instrumentations in 59% (13% percutaneous vs. 87% open instrumentation). Regarding the location of the surgeries, 60% were performed on the lumbar spine, 26% on the cervical, and 26% on the thoracic spine.

The mean blood loss was 638 ml (range: 0–8000 ml). Thereby, the main diagnosis tumour correlated with higher blood loss (mean: 1149 ml within this subgroup).

### Complications and risk factors

Within our study collective, intraoperative complications emerged in 26 cases (4.9%). These included accidental Dural lesions (19 patients), injury of the vertebral artery (3 patients), premature termination of surgery due to unexpectedly high blood loss or cardiopulmonary instability (2 patients), accidental rhizotomy (1 patient), and cement leakage (1 patient).

Postoperatively, 135 (25.7%) patients suffered complications within 30 days. Of those, 83 were surgical complications and 52 were systemic. Interestingly, 19% of these patients had already been discharged when the complications occurred (median time of discharge to complication: 0.5 days [IQR 0–8.5 days]). The complications are recorded in Table 2. Risk factor analysis is presented in Table 3.

**Table 2:** Complication rates in % according to their frequency.

	No	% (of all pat)
**Intraoperative complications**		
CSF leakage	19	3.6%
Injury of vertebral artery	3	0.6%
Premature termination	2	0.4%
Nerve root damage	1	0.2%
Cement leakage	1	0.2%
	**26**	**4.9%**
**30-day postoperative complications**		
Surgical		
Rebleeding	16	3.0%
Wound healing disorder	11	2.1%
Motor deficit	10	1.9%
CSF leakage	10	1.9%
Screw mispositioning	7	1.3%
Abscess/spondylodiscitis	6	1.1%
New dysesthesia	6	1.1%
New back pain	5	1.0%
New leg pain	4	0.8%
Persistent stenosis	4	0.8%
Material failure	4	0.8%
	**83**	**15.8%**
Systemic		
Urinary tract infection	13	2.5%
Pneumonia	7	1.3%
Cardiac event	6	1.1%
Pulmonary embolism	6	1.1%
Renal insufficiency	3	0.6%
Blood stream infection	3	0.6%
Dysphagia	3	0.6%
Psychiatric disorders	3	0.6%
Other	8	1.5%
	**52**	**9.9%**

Significant values are in [bold].

**Table 3:** Risk factor analysis.

	Intraoperative complications				Postoperative complications				Revision surgeries			
	OR	IQR	p	sig	OR	IQR	p	sig	OR	IQR	p	sig
**Patient characteristics**												
Age	0.99	0.97–1.01	0.41	ns	1.01	1.00–1.03	0.07	ns	1.00	0.98 –1.02	0.91	ns
BMI	0.99	0.93 –1.05	0.77	ns	1.02	0.98 –1.05	0.43	ns	1.07	1.02 –1.13	**0.004**	**
ASA score	0.80	0.47 –1.36	0.41	ns	0.99	0.70 –1.38	0.93	ns	0.94	0.58 –1.52	0.80	ns
KPS postoperative	0.17	0.01 –4.60	0.29	ns	0.03	0.00 –0.81	**0.04**	*	0.91	0.02 –43.60	0.96	ns
KPS preoperative	1.73	0.02 –161	0.81	ns	21.43	0.45 –1016	0.12	ns	0.11	0.00 –12.20	0.36	ns
mRS postoperative	0.66	0.34 –1.27	0.21	ns	1.39	0.78 –2.48	0.26	ns	1.78	0.83 –3.83	0.14	ns
mRS preoperative	1.90	0.92 –3.93	0.08	ns	0.88	0.48 –1.60	0.67	ns	0.61	0.27 –1.37	0.23	ns
**Co-morbidities**												
Charlson Index	0.96	0.73–1.27	0.79	ns	0.90	0.74–1.09	0.27	ns	1.09	0.85–1.40	0.51	ns
Charlson Index age-adjusted	1.03	0.81–1.30	0.82	ns	1.17	0.99–1.39	**0.01**	*	0.98	0.78–1.22	0.83	ns
Diagnosis count	0.91	0.75–1.09	0.30	ns	1.02	0.91–1.13	0.75	ns	1.10	0.95–1.26	0.20	ns
Depression		χ^2^ = 1.71	0.19	ns		χ^2^ = 0.15	0.70	ns		χ^2^ = 0.87	0.35	ns
Tumour with metastases		χ^2^ = 0.05	0.82	ns		χ^2^ = 6.21	**0.01**	*		χ^2^ = 4.75	**0.03**	*
Atrial fibrillation		χ^2^ = 0.44	0,51	ns		χ^2^ = 5.67	**0.02**	*		χ^2^ = 2.14	0.14	ns
**Laboratory results**												
Creatinine pre-OP	0.15	0.02–0.90	**0.04**	*	1.04	0.95–1.15	0.36	ns	0.46	0.14–1.47	0.19	ns
GFR pre-OP	0.98	0.96–1.00	0.08	ns	0.99	0.98–1.00	0.06	ns	0.99	0.97–1.00	0.10	ns
CRP pre-OP	1.02	0.97–1.06	0.49	ns	1.02	0.99–1.05	0.30	ns	1.03	0.99–1.07	0.10	ns
Quick pre-OP	0.98	0.96–1.01	0.12	ns	0.99	0.97–1.01	0.30	ns	0.98	0.96–1.00	**0.04**	*
PTT pre-OP	0.98	0.90–1.07	0.70	ns	0.98	0.93–1.04	0.51	ns	0.99	0.92–1.06	0.81	ns
Platelets pre-OP	1.00	1.00–1.00	0.64	ns	1.00	1.00–1.00	0.19	ns	1.00	1.00–1.00	0.49	ns
Haemoglobin post OP	0.92	0.76–1.12	0.43	ns	0.81	0.71–0.92	**0.001**	***	0.95	0.80–1.12	0.55	ns
Haemoglobin pre-OP	1.09	0.89–1.33	0.42	ns	1.20	1.05–1.37	**0.01**	**	1.11	0.93–1.33	0.23	ns
**Surgery specific factors**												
Duration of surgery	1.00	1.00–1.01	**0.03**	*	1.00	1.00–1.01	**0.002**	**	1.00	1.00–1.01	0.20	ns
Spinal levels of surgery	1.32	0.97–1.81	0.08	ns	0.73	0.56–0.97	**0.03**	*	0.68	0.50–0.92	**0.01**	*
Blood loss	1.00	1.00–1.00	**0.01**	**	1.00	1.00–1.00	0.81	ns	1.00	1.00–1.00	**0.05**	*
Instrumentation	2.00	0.44–9.15	0.37	ns	1.25	0.43–3.60	0.68	ns	1.06	0.42–2.70	0.90	ns
Use of cement	0.70	0.16–3.06	0.64	ns	0.95	0.29–3.17	0.93	ns	0.95	0.30–2.98	0.92	ns
**Inpatient factors**												
Discharge not home	0.60	0.30–1.23	0.16	ns	1.16	0.75–1.82	0.50	ns	0.96	0.63–1.46	0.86	ns
Length of stay	1.03	0.99–1.07	0.17	ns	1.05	1.01–1.09	**0.02**	*	1.03	1.01–1.05	**0.001**	***
Transfusion count	1.06	0.65–1.73	0.82	ns	0.82	0.55–1.23	0.35	ns	1.22	0.86–1.72	0.27	ns

Significant values are in [bold].

Furthermore, 61 patients (12%) required revision surgery within 30 postoperative days (median time to revision surgery: 11 days [IQR 5–15 days]).

### Intraoperative complications

The multivariate logistic regression analysis revealed blood loss (p = 0.01, OR = 1.0) and duration of surgery (p = 0.03, OR = 1.0) as independent risk factors for intraoperative complications.

The spinal instrumentation (p = 0.37), previous spine surgeries (p = 0.45), or other patient-specific risk factors did not influence the rate of intraoperative complications.

### Postoperative complications

Concerning postoperative complications, the following patient-specific risk factors could be identified: postoperative KPS (p = 0.04, OR = 0.03) and CCI age-adjusted (p = 0.01, OR = 1.17). Patient age showed borderline significance (p = 0.069, OR = 1.01).

The relevant blood parameters were haemoglobin concentration postoperative (p = 0.001, OR = 0.81) and haemoglobin preoperative (p = 0.01, OR = 1.20).

Analysis of surgery itself revealed that spinal levels of surgery (p = 0.03, OR = 0.73) and the duration of surgery are strongly correlated with postoperative complications (p = 0.001, OR = 1.0).

Furthermore, the duration of hospital stay (p = 0.02, OR = 1.05) was strongly associated with higher complication rates. The median length of hospital stay was double in the case of complications: 15 days (IQR 6–23) with complications; 7 days (IQR 3–12) without complications. Complication rates according to discharge destination and frequency were: home (21% complications), another department at the same hospital (20% complications), another hospital (38% complications), rehabilitation centre (35% complications) and death during follow up (100% complications).

Surgeries combining cervical and thoracic spine or thoracic and lumbar spine treatments showed significantly more postoperative complications than interventions on the cervical, thoracic, or lumbar alone. The use of navigation devices showed no significant influence on postoperative complications.

Patients diagnosed with metastatic tumour (p = 0.01, χ^2^ = 6.21) or atrial fibrillation (p = 0.02, χ^2^ = 5.67) developed significantly more postoperative complications.

### Revision surgeries

Concerning revision surgeries, patients with higher BMI underwent revision surgeries more often (p = 0.004, OR = 1.07). Other patient-specific risk factors were not predictive for revision surgeries.

Confirming the results of postoperative complications, the more spinal levels were operated on (spinal levels of surgery p = 0.01, OR = 0.68) and the more blood loss occurred (blood loss p = 0.05, OR = 1.0) the more revision surgeries had to be performed.

Length of hospital stays also correlated with the number of revision surgeries (p = 0.001, OR = 1.03).

Patients with metastatic tumours also were revised more often (p = 0.03, χ^2^ = 4.75).

**Figure 2:**
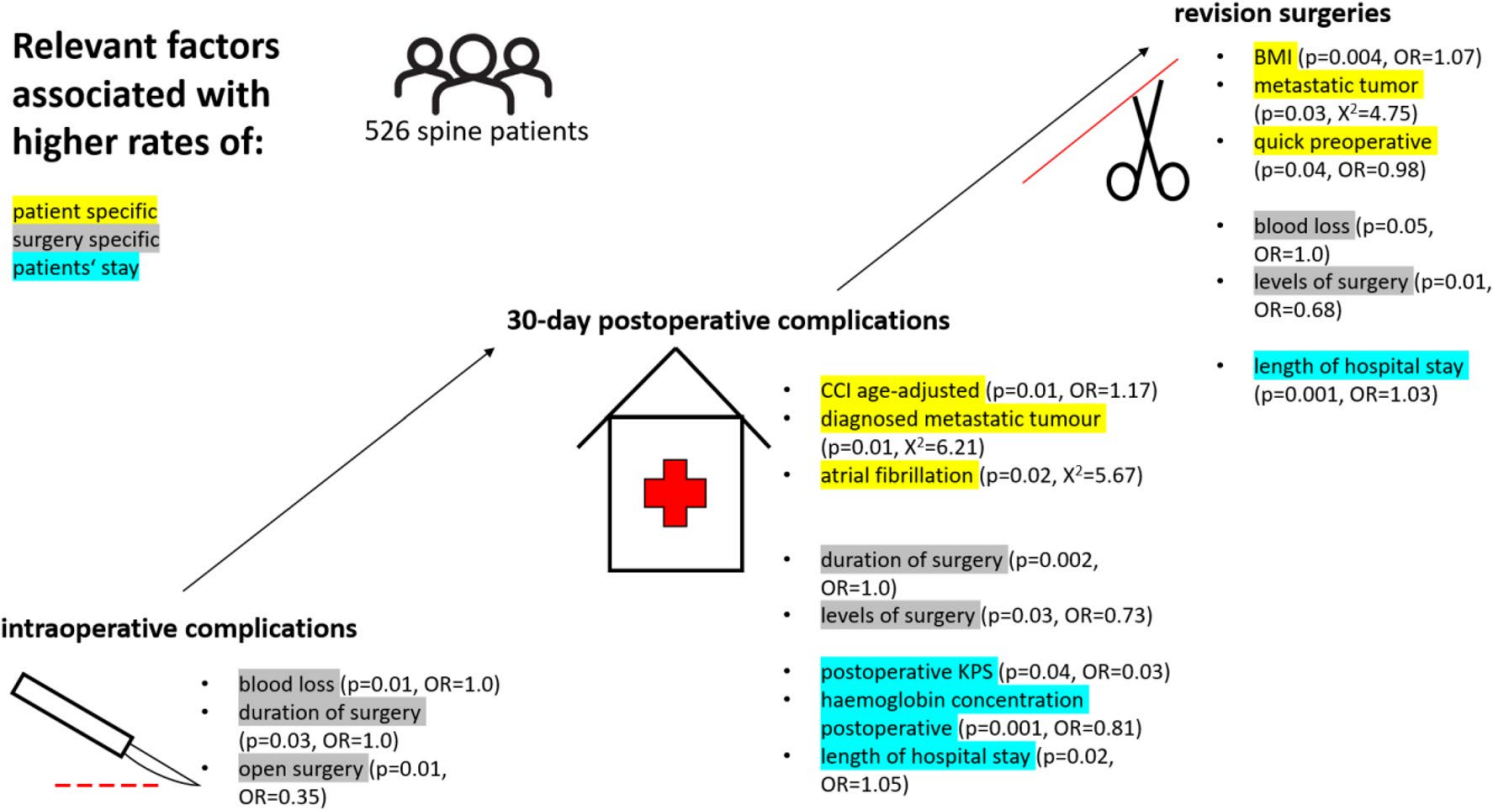
Significant risk factors for complications during patients’ hospital stay.

Figure 2 provides an overview of the significant parameters.

We graded the complications in this study into “major”—requiring revision surgery or leading to transferal to intensive care unit and “mild” for complications that could be resolved completely within days after surgery.

Interestingly, there is no significant difference regarding age within the groups. Mean age in the group with mild complications was 68 years, in the group of major complications it was 65 years, respectively. According to the previous presented results, analysis showed that the group of major complications showed higher BMI values than did the mild complication group (mean 29.1 versus 25.6).


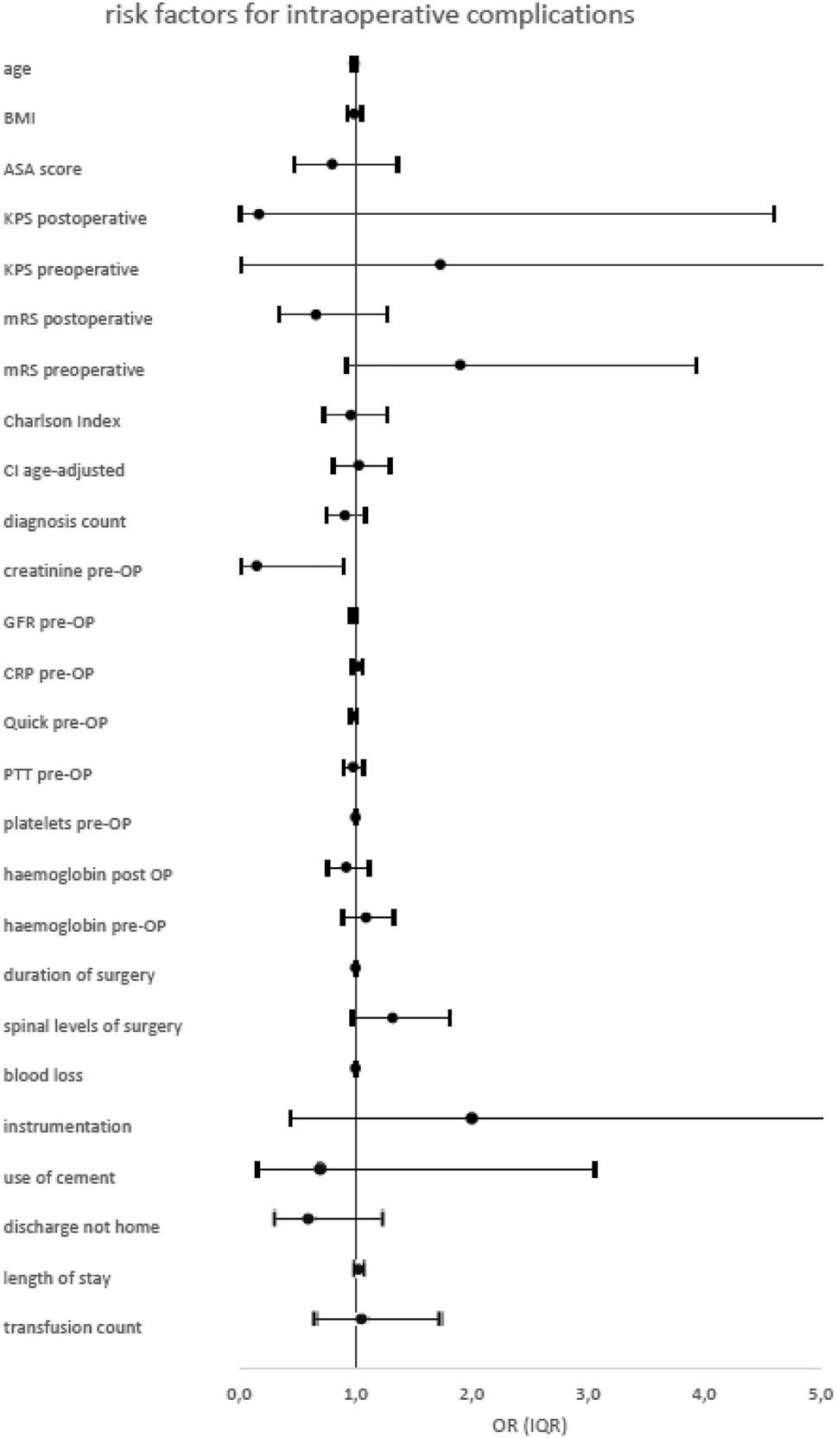



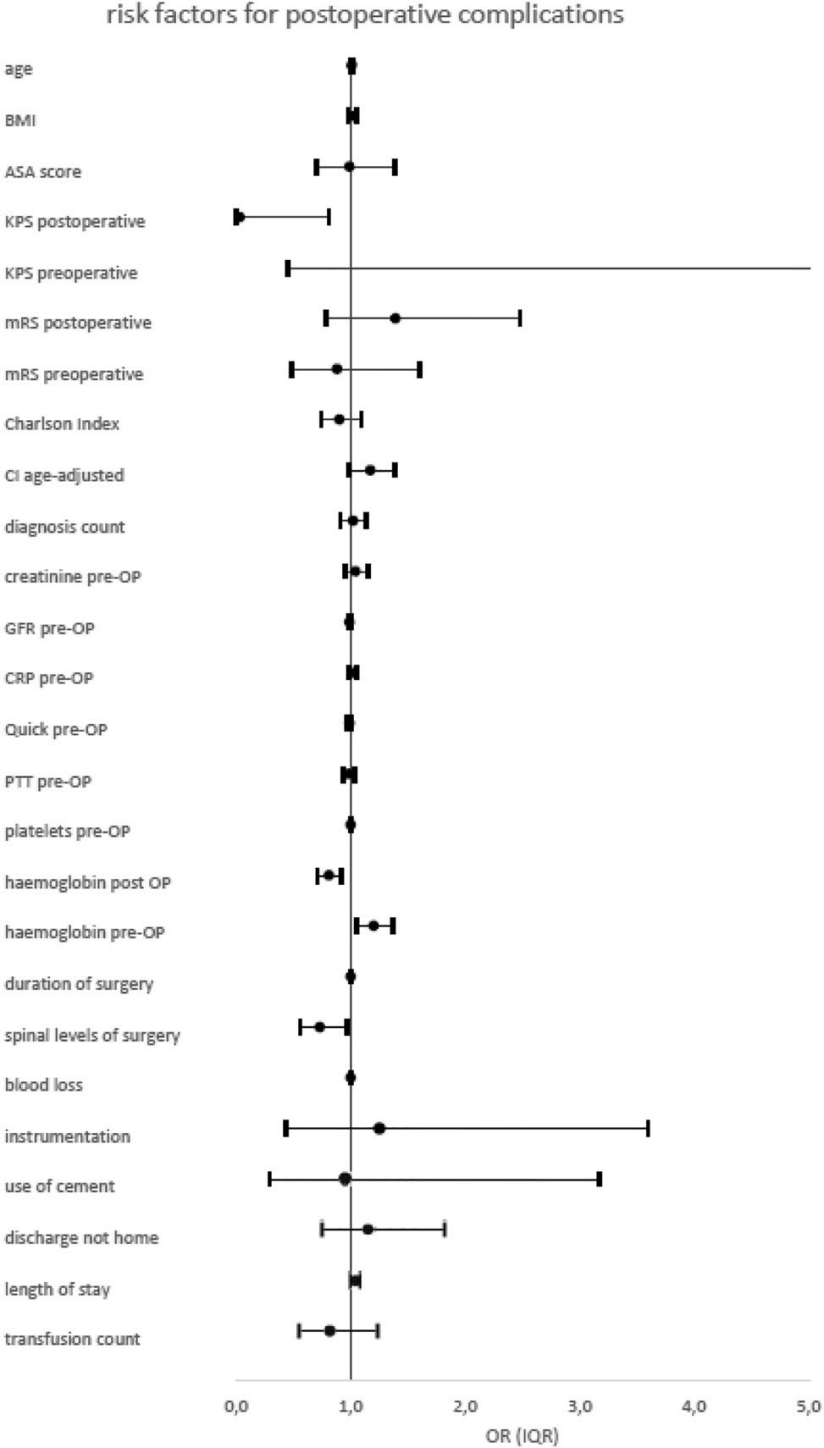



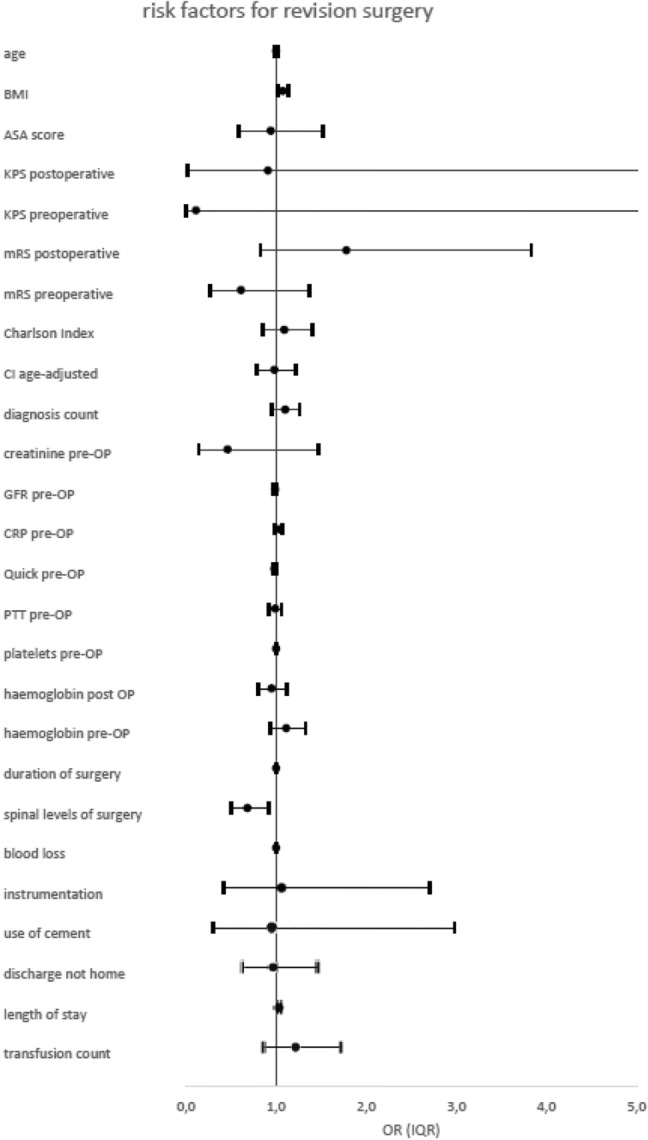


## Discussion

In this prospective single-centre observational study of 526 patients, complications and outcomes during and after spinal surgeries were analysed.

### Patient-specific risk factors

The patients’ median age in our collective of spinal surgeries was 67 years (range: 13–96). Thereby, being older did not correlate with more intra- or postoperative complications nor to the probability of undergoing revision surgeries. However, age-adjusted CCI showed a significant correlation with postoperative complications (p = 0.01), though CCI did not (p = 0.27). This modern series of patients treated at a spine centre highlight that age in isolation is no longer a significant risk factor. Earlier, we could show that neurosurgical procedures offer favourable outcomes and complication rates in patients over 80 and 90 years old^10–13^. Outcomes in those patients were also favourable in this study, showing an improvement in both SF-12 PCS and MCS, as well as in postoperative ODI scores after 3 and 12 months. Age-adjusted CCI in this study reflected a median of 4 (IQR 3–6) and an expected one-year mortality in 52% cases, whereas mortality during a one-year follow-up in this study was only 9.1%^7^. This shows a far better outcome despite the multiple comorbidities in our patient collective. In addition, we could not record the complications in the elderly to be more severe then in the younger collective. Also, patients with a higher BMI underwent more revision surgeries in our patient collective (p = 0.004, OR = 1.07). These pertained to wound revisions, material failures, and rebleeding in particular. This highlights that the comorbidities of the patients, as well as their constitution, should be considered, whereas patients’ age is no longer relevant.

It is known that patients with more comorbidities tend to stay longer in hospitals^14–16^. Hospitalization could hence be shown as an independent risk factor for complications, independent of operative or conservative therapies^11^. In this study, the length of hospital stay was also associated with postoperative complications (p = 0.02) as well as with probability of requiring revision surgeries (p = 0.001). As expected, hospital stays of the elderly is much longer, then in the younger patients, resulting in higher complication rates at the end. Furthermore, discharge to a location that is not home seems to increase complication rates postoperatively. Attempts should hence be made for early discharge to home, especially in the old patient collective, guaranteeing early mobility and independence of the patients to prevent complications, readmission, and revision surgeries. Other studies have confirmed that early discharge is not associated with higher complication rates after a diversity of operative procedures^17–21^.

Another relevant risk factor for postoperative complications in this analysis was a low postoperative KPS (p = 0.04) triggering more problems in the patient, alongside reduced mobility and self-sufficiency. Lower postoperative KPS could be shown earlier as an independent predictor for overall complications, overall survival, and outcomes for patients with spinal metastases^22–25^.

### Co-morbidities

In this analysis, postoperative complication rates were observed to be independently influenced by the following co-morbidities of the patients: metastatic tumour (p = 0.01) and atrial fibrillation (p = 0.02). Thus, patients with metastatic tumours also exhibited higher rates of revision surgeries (p = 0.03). Other common risk factors as BMI, ASA score, nicotine or alcohol abuse, as well as co-medication showed no significance in this study collective.

In patients with atrial flutter, the most frequent complications were re-bleeding (24%) and wound healing disorders (12%), whereas their anticoagulant medication was stopped perioperatively in most of the cases. Most of the studies show atrial fibrillation as postoperative complications after cardiac and noncardiac surgery, instead of leading to postoperative complications^31–33^. Patients with atrial fibrillation showed a median age of 79 years and a BMI of 29 which could also explain their higher rates of complications, as in this study, a higher BMI led to higher revision rates (p = 0.004, OR = 1.07).

As per the estimation, metastatic tumours lead to higher rates of postoperative complications, as well as revision rates.

### Laboratory results

Perioperative anaemia is known to increase complications and morbidity rates^40–42^. It leads to longer hospital stays and minor outcomes in the 30-day period after elective spine surgery, whereas in other studies, preoperative anaemia could not be correlated to postoperative complication rates in spine surgery^42–45^. In this study, patients with postoperative anaemia showed longer hospital stays (median 9 days versus 2 days without anaemia) and significantly higher risks of postoperative complications (p = 0.001). In our study, preoperative anaemia could also be correlated with postoperative complications (p = 0.01). Screening for and therapy of anaemia before elective spine surgery, as well as postoperatively, is therefore recommended^46^. The rate of revision surgeries was not associated with haemoglobin concentrations.

### Surgery specific factors

As expected, duration of surgery and blood loss during surgery were strongly correlated with intraoperative and postoperative complications as well as with revision surgeries. Many studies have confirmed this correlation^47–49^. No other surgery-specific factors could significantly influence the complication rates. The use of different navigation devices had no impact on the outcomes, though we could show earlier a significant reduction in the mispositioning of pedicle screws through 3D navigation devices^50^.

## Conclusions

An analysis of a consecutive cohort of 526 spinal cases of a high output spine centre shows optimal outcomes despite a challenging patient collective. Age with a median of 67 years, as well as the high rate of patients with preceding spine surgeries (rate of 35%), reflects the new challenges in modern neurosurgical departments. In this light, revision rates of 12%, as well as intra- and postoperative complications were analysed.

In patient selection, as patients with a higher BMI showed higher odds for revision surgeries, indications for obese patients should be considered more critically. Efforts to reduce the duration of surgeries, alongside reduced blood loss, can reduce patients’ complications, as confirmed through this study. After surgery, prevention of postoperative anaemia and early mobilization and discharge of the patients should be ensured, wherever possible.
